# History matters...through partnerships that advance research, education, and public service

**DOI:** 10.5195/jmla.2017.213

**Published:** 2017-07-01

**Authors:** Jeffrey S. Reznick, E. Thomas Ewing

In the summer of 2015, as part of a seminar made possible through a grant to Virginia Polytechnic Institute and State University (Virginia Tech) from the National Endowment for the Humanities (NEH), a dozen primary and secondary teachers from across the United States gathered at the National Library of Medicine (NLM) in Bethesda, Maryland. Joining them and offering a presentation about the significance of the history of Spanish influenza in recent scientific research was the distinguished epidemiologist Jeffery Taubenberger of the National Institute for Allergy and Infectious Diseases (NIAID), National Institutes of Health (NIH), whose research has transformed modern scientific understanding of influenza. Complementing Taubenberger’s presentation, NLM historians and librarians guided the group in studying firsthand a variety of rare and unique primary sources from the library’s historical collections—medical publications, newspapers, original documents, and ephemera—all dating from the 1918 influenza pandemic, and many of which, due to their physical condition could not yet be digitized and made available remotely in electronic form.

**Figure 1:**
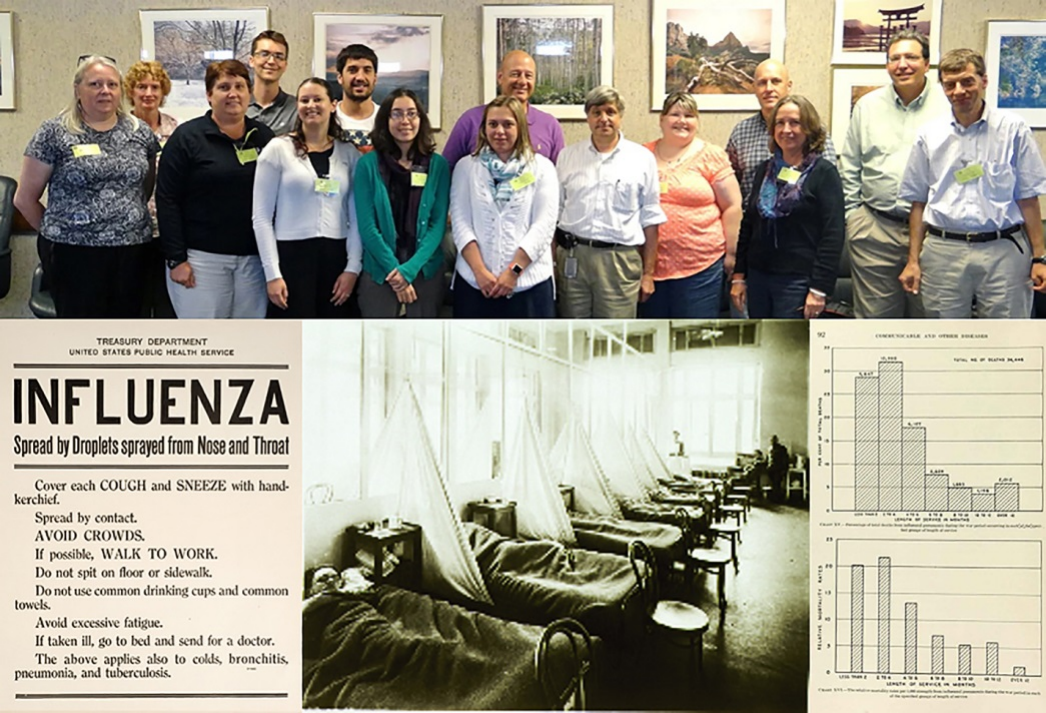
Seminar participants and materials

Figure 1 shows the seminar participants, in a photograph by Wilsonia Cherry, deputy director, NEH Division of Education Programs, including Taubenberger (front row, sixth from the left). Below this photograph is a selection of NLM collection items that the group studied, including, right to left, a broadside about influenza, which recommended ways to prevent its spread; a photograph of Influenza Ward 1 in US Army Camp Hospital number 45, Aix-les-Bains, France; and contemporary charts that show death rates of soldiers from influenza.

Complementing this program at NLM were daylong visits by the group to the Library of Congress and the National Archives, as well as a lecture at Virginia Tech by Nancy K. Bristow, professor of history at University of Puget Sound and author of the book *American Pandemic.*

While the traditional relationship between a library and a university shaped this program—each working with the other in mutually supportive ways to provide direct access to and expertise to interpret valuable research materials—the seminar at NLM offered much more. It leveraged the association of NLM to NIH—indeed, the library’s very physical location on the NIH campus—to connect directly to this group of teachers one of the world’s most accomplished and well-known researchers, who offered them enlightening, inspiring, and thoughtful perspectives on the importance of medical history for current medical research.

This NEH summer seminar marked a major chapter in the longstanding and multifaceted collaboration between NLM’s History of Medicine Division and Virginia Tech’s Department of History, and in the engagement of both organizations with NEH. Together, these relationships, combined with many others, have yielded a substantial and growing record of advancing research, education, and public service [1, 2].

The collaboration between NLM’s History of Medicine Division and Virginia Tech’s Department of History began in 2012, when Brett Bobley, director of the NEH Office of Digital Humanities, introduced the current authors, thinking that we would have common interests in research, teaching, and public service. Our work together blossomed during the months that followed, in large part through our participation in “Shared Horizons: Data, Biomedicine, and the Digital Humanities,” an NEH-funded international symposium held in April 2013 at the University of Maryland, cosponsored by NLM and Research Councils UK. Shortly thereafter, Ewing gave an invited lecture at NLM, and this engagement led to our institutions cosponsoring “An Epidemiology of Information: New Methods for Interpreting Disease and Data,” another NEH-funded symposium, held in October 2013 at the Virginia Tech Research Center in Arlington. This symposium explored new methods for large-scale data analysis of epidemic disease, involving Reznick in the research forum, along with Virginia Tech social sciences librarian Bruce Pencek, who served as one of the coprincipal investigators of the project, in cooperation with faculty from Virginia Tech’s Departments of Computer Science and English.

A year later, in October 2014, we copresented a paper on our research and institutional collaborations at the annual “Big Data Humanities” conference of the Institute of Electrical and Electronics Engineers (IEEE) and coauthored an article in the proceedings of the conference [3]. That same year, a group of Virginia Tech undergraduates visited NLM to study the history of the Russian influenza (1889–1890) through a variety of primary sources and to meet, learn from, and be inspired by Taubenberger and his colleague David Morens, senior advisor to the director of NIAID. This experience empowered these students to present their research to a broader audience through a series of postings on *Circulating Now*, the popular blog of NLM’s History of Medicine Division. Interested readers can learn more about the details of these postings via Ewing’s research site. In contributing these postings to *Circulating Now,* the student-researchers joined their firsthand experiences in NLM’s historical collections—exploring the life of an Irish doctor, understanding the geographic spread of the disease, and interpreting news coverage of the outbreak—with the experiences of dozens of others who have written for the blog—including scholars, educators, and students—resulting in a rich record of engagement with the historical collections of the world’s largest biomedical library.

A year later, another group of Virginia Tech students, working this time on the history of tuberculosis in Virginia, also had opportunities to publish their research completed at NLM, including studies of public responses to Robert Koch’s research on tuberculosis, methods of advertisers who exploited fears about this disease, and early uses of data processing techniques to calculate numbers of victims of this disease.

The year 2015 was equally fruitful for our collaboration. We spent much of the year envisioning a landmark workshop that would gather together a new generation of researchers to explore and apply the tools of the digital humanities to historical-medical research questions. That winter, at the Virginia Tech Research Center in Arlington, a panel of medical historians, including Reznick and his NIH/NIAID colleague Morens, convened to offer expert feedback on research that a group of Virginia Tech undergraduates completed on the Russian influenza as a global pandemic. The following spring, thanks to the generous support of the NEH Office of Digital Humanities, through a cooperative agreement with Virginia Tech as well as generous support of the Wellcome Trust, the workshop we envisioned, “Images and Texts in Medical History: An Introduction to Methods, Tools, and Data from the Digital Humanities,” became a reality when we convened it, to much public acclaim, at the NIH Natcher Conference Center.

That summer our collaboration grew further, with cosponsorship of the American Historical Association (AHA), when Reznick represented NLM on another panel of public scholars, which included Katherine Ott, curator, Division of Medicine and Science of the National Museum of American History, and Christine Sizemore, chief, Tuberculosis, Leprosy and Other Mycobacterial Diseases Section, NIAID. Together, they offered critical feedback on research that Virginia Tech undergraduates completed about tuberculosis in American history. This research itself, based substantially on NLM’s historical collections, resulted in two postings for *AHA Today,* the official blog of AHA, an outcome that, like *Circulating Now,* spotlighted rigorous and primary source–based historical research that these undergraduates completed and their ability to communicate this research effectively to a broad public audience. Bringing our collaboration both full-circle and leveraging it to have even greater scholarly and educational value, the syllabus of Ewing’s course “Introduction to Data in Social Context” includes as required readings the postings that the now former Virginia Tech undergraduates contributed both to *Circulating Now* and *AHA Today*.

The NLM/Virginia Tech collaboration has flourished in a culture of mutual trust and support, both individually and institutionally, as we navigated expectations of the fields of higher education and public service, with specific and sustained attention to the importance of developing transferable skills among students, moving rigorous scholarship and related learning outcomes out of classrooms, and establishing and supporting public engagement with complex problems. Collaborations like ours make great sense strategically, financially, and managerially, as they help focus human and materials resources, foster creativity and teamwork, engage colleagues, and expand learning and professional networks. Collaborations like ours also make great sense because they make history matter, by unlocking the richness of the global medical historical record for the benefit of researching, teaching about, and learning about the human condition.
